# Supplementary material for: Omega-6 Polyunsaturated Fatty Acids Enhance Tumor Aggressiveness in Experimental Lung Cancer Model: Important Role of Oxylipins
Source: Int J Mol Sci. 2022 May 31;23(11):6179. doi: 10.3390/ijms23116179 (PMC9181584; doi:10.3390/ijms23116179)
Supplement: Supplementary file 1 [file ijms-23-06179-s001.zip › ijms-1713947-supplementary.pdf]

Supplementary Table S1. w-6 oxylipins levels in plasma of mice with balanced and w-6 rich diet. M±SEM, *p<0.05, **p<0.01, ***p<0.001

ω-6 PUFAs	Enzyme	Oxilypins	Balanced diet (μmol/L)	ω-6 rich diet (μmol/L)	*P value* (t-test)*
**LA**	**COX**	---	---	---	---
	**LOX**	9-HODE	2654.3±174.1	10285±612.4	***
		13-HODE	2097.4±112-5	8985.5±473.2	***
		9,10,13-TriHOME	112.9±38.5	152.3±15.9	ns
		9,12,13-TriHOME	192.9±63.2	276.9±33.7	ns
	**CYP450**	9,10-EpOME	829.5±229.9	1150.9±341.1	ns
		12,13-EpOME	654.8±195.3	995.2±311.0	ns
		9,10-DiHOME	11.3±3.5	13.5±3.0	ns
		12,13-DiHOME	13.1±3.4	21.0±11.1	ns
**ARA**	**COX**	TXB_2	11.0±3.5	28.2±9.4	*
		PGD_2	0.2±0.06	0.5±0.1	*
		PGF_{2a}	1.0±0.2	2.1±0.7	ns
		PGE_2	0.3±0.04	0.6±0.2	ns
	**LOX**	LXA_4	3.0±1.2	7.9±2.5	*
		LTB_4	0.7±0.3	1.5±0.6	ns
		6-trans-LTB_4	0.3±0.09	0.5±0.4	ns
		5-oxo-ETE	20.9±4.7	56.7±5.1	ns
		12-oxo-ETE	142.5±60.4	534.6±177.1	*
		15-oxo-ETE	7.9±1.9	17.1±5.7	ns
		5-HETE	22.2±8.8	29.7±8.7	ns
		8-HETE	11.6±3.5	24.9±7.2	ns
		9-HETE	0.01±0.001	1.2±0.4	***
		11-HETE	17.8±6.4	38.0±14.6	ns
		12-HETE	325.1±201.1	1751.8±400.1	**
		15-HETE	22.9±7.9	48.9±17.7	ns
	**CYP450**	20-HETE	0.2±0.04	0.3±0.06	ns
		5,6-EpETrE	932.2±207.2	1431.2±418.8	ns
		8,9-EpETrE	412.2±144.1	583.6±156.7	ns
		11,12-EpETrE	197.1±43.1	271.7±75.3	ns
		14,15-EpETrE	130.9±27.6	183.4±52.0	ns
		5,6-DiHETrE	0.6±0.1	1.2±0.1	**
		8,9-DiHETrE	0.7±0.2	1.6±0.1	*
		11,12-DiHETrE	0.4±0.1	0.8±0.1	ns
		14,15-DiHETrE	0.8±0.2	1.5±0.2	*

Supplementary Table S2. w-3 oxylipins levels in plasma of mice with balanced and w-6 rich diet. M±SEM, *p<0.05, **p<0.01, ***p<0.001

ω-3 PUFAs	Enzyme	Oxilypins	Balanced diet (μmol/L)	ω-6 rich diet (μmol/L)	*p value*
**ALA**	**COX**	---	---	---	---
	**LOX**	9-HOTrE	7.4±2.5	6.3±1.0	ns
		13-HOTrE	7.6±1.6	0.2±0.03	**
	**CYP450**	9,10-EpODE	55.9±22.0	18.0±4.5	ns
		12,13-EpODE	45.7±18.1	14.8±3.8	ns
		15,16-EpODE	112.5±46.9	43.1±10.1	ns
		9,10-DiHODE	0.3±0.1	0.2±0.1	ns
		12,13-DiHODE	2352.3±718.6	1686.7±239.4	ns
		15,16-DiHODE	2.0±0.2	2.4±0.2	ns
**EPA**	**COX**	PGD_3	0.3±0.1	0.08±0.02	*
	**LOX**	5-HEPE	42.3±19.5	4.1±1.4	*
		8-HEPE	33.4±12.4	1.5±0.7	*
		12-HEPE	373.5±204.1	103.5±14.5	ns
		15-HEPE	73.4±33.2	10.0±2.7	*
	**CYP450**	8,9-EpETE	131.7±34.4	5.2±1.7	**
		11,12-EpETE	106.7±27.1	6.0±1.2	**
		14,15-EpETE	184.4±44.0	10.2±1.7	**
		17,18-EpETE	226.2±55.8	14.4±3.0	***
		8,9-DiHETE	1.0±0.1	0.9±0.4	ns
		11,12-DiHETE	0.6±0.05	0.09±0.02	***
		14,15-DiHETE	0.8±0.1	0.1±0.02	**
		17,18-DiHETE	3.9±0.7	1.4±0.2	**
**DHA**	**COX**	---	---	---	---
	**LOX**	17-HDoHE	786.3±412.2	240.9±39.8	ns
	**CYP450**	7,8-EpDPE	5353.7±1520.7	1207.2±319.2	*
		10,11-EpDPE	398.5±117.1	91.0±22.6	*
		13,14-EpDPE	256.5±74.3	58.2±14.5	*
		16,17-EpDPE	253.3±79.4	54.4±13.7	*
		19,20-EpDPE	325.1±102.2	61.0±17.2	*
		4,5-DiHDPE	6.1±3.7	1.4±0.2	ns
		7,8-DiHDPE	4550.1±771.2	463.9±165.8	**
		10,11-DiHDPE	1.2±0.2	0.4±0.1	*
		13,14-DiHDPE	1.0±0.2	0.4±0.02	*
		16,17-DiHDPE	2.0±0.4	0.7±0.09	*
		19,20-DiHDPE	17.7±7.1	4.9±0.4	*
